# Managing Neonatal and Early Childhood Syndromic Sepsis in Sub-District Hospitals in Resource Poor Settings: Improvement in Quality of Care through Introduction of a Package of Interventions in Rural Bangladesh

**DOI:** 10.1371/journal.pone.0170267

**Published:** 2017-01-23

**Authors:** Ahmed Ehsanur Rahman, Afrin Iqbal, D. M. Emdadul Hoque, Md. Moinuddin, Sojib Bin Zaman, Qazi Sadeq-ur Rahman, Tahmina Begum, Atique Iqbal Chowdhury, Rafiqul Haider, Shams El Arifeen, Niranjan Kissoon, Charles P. Larson

**Affiliations:** 1 Maternal and Child Health Division, International Centre for Diarrhoeal Disease Research, Bangladesh (icddr,b), Dhaka, Bangladesh; 2 The Department of Pediatrics, The University of British Columbia, Vancouver, British Columbia, Canada; Centre Hospitalier Universitaire Vaudois, FRANCE

## Abstract

**Introduction:**

Sepsis is dysregulated systemic inflammatory response which can lead to tissue damage, organ failure, and death. With an estimated 30 million cases per year, it is a global public health concern. Severe infections leading to sepsis account for more than half of all under five deaths and around one quarter of all neonatal deaths annually. Most of these deaths occur in low and middle income countries and could be averted by rapid assessment and appropriate treatment. Evidence suggests that service provision and quality of care pertaining to sepsis management in resource poor settings can be improved significantly with minimum resource allocation and investments. Cognizant of the stark realities, a project titled ‘Interrupting Pathways to Sepsis Initiative’ (IPSI) introduced a package of interventions for improving quality of care pertaining to sepsis management at 2 sub-district level public hospitals in rural Bangladesh. We present here the quality improvement process and achievements regarding some fundamental steps of sepsis management which include rapid identification and admission, followed by assessment for hypoxemia, hypoglycaemia and hypothermia, immediate resuscitation when required and early administration of parenteral broad spectrum antibiotics.

**Materials and Method:**

Key components of the intervention package include identification of structural and functional gaps through a baseline environmental scan, capacity development on protocolized management through training and supportive supervision by onsite ‘Program Coaches’, facilitating triage and rapid transfer of patients through ‘Welcoming Persons’ and enabling rapid treatment through ‘Task Shifting’ from on-call physicians to on-duty paramedics in the emergency department and on-call physicians to on-duty nurses in the inpatient department.

**Results:**

From August, 2013 to March, 2015, 1,262 under-5 children were identified as syndromic sepsis in the emergency departments; of which 82% were admitted. More neonates (30%) were referred to higher level facilities than post-neonates (6%) (p<0.05). Immediately after admission, around 99% were assessed for hypoxemia, hypoglycaemia and hypothermia. Around 21% were hypoxemic (neonate-37%, post-neonate-18%, p<0.05), among which 94% received immediate oxygenation. Vascular access was established in 78% cases and 85% received recommended broad spectrum antibiotics parenterally within 1 hour of admission. There was significant improvement in the rate of establishing vascular access and choice of recommended first line parenteral antibiotic over time. After arrival in the emergency department, the median time taken for identification of syndromic sepsis and completion of admission procedure was 6 minutes. The median time taken for completion of assessment for complications was 15 minutes and administration of first dose of broad spectrum antibiotics was 35 minutes. There were only 3 inpatient deaths during the reporting period.

**Discussion and Conclusion:**

Needs based health systems strengthening, supportive-supervision and task shifting can improve the quality and timeliness of in-patient management of syndromic sepsis in resource limited settings.

## Introduction

Sepsis is defined as a dysregulated systemic inflammatory response due to a suspected or proven infection which can lead to tissue damage, organ failure, and death [1, 2]. It is the final common pathway to death due to pneumonia, diarrhoeal disease, malaria, bacterial septicemia, dengue and severe nosocomial infections among others [3]. With an estimated 30 million cases per year, it is a global public health concern [4, 5]. Severe infections leading to sepsis accounts for more than half of all under five deaths and around one quarter of all neonatal deaths annually [6–8]. The proportionate contribution of sepsis and other severe infections among neonatal deaths is greater than preterm birth (15%) and intra-partum related complications (11%) [6]. However, until recently, sepsis remained as a neglected issue in the global agenda. As a consequence, the rate of reduction of sepsis specific mortality between 1990 and 2010 has been much slower (4%) than that of preterm birth (29%) and intra-partum related complications (20%) [9]. Out of the total global research spending on newborn health, less than 15% is spent on prevention and treatment of severe infections and sepsis [10].

Early childhood sepsis, particularly neonatal sepsis, is among the most critical concerns for low and middle income countries. In 2012, an estimated 6.1 million neonates suffered from syndromic sepsis in South Asia and Sub Saharan Africa [11]. A significant proportion of these cases did not survive as the case fatality rate is very high with significant variation between countries and regions (8%-80%) [12, 13]. Similarly in Bangladesh, a developing country in South Asia, sepsis and other severe infections account for 39% of all under five deaths and 37% of all neonatal deaths [14].

Sepsis should be recognized early and treated aggressively in hospital settings as appropriate case management can prevent 84% of deaths due to sepsis [15, 16]. In adults, each hour of delay in administration of appropriate antimicrobial agents in severe sepsis decreases survival rate by 7.6% [17]. While similar data in children are lacking, quality improvement endeavours which include early administration broad spectrum antibiotics and supportive care through a resuscitation bundle have resulted in dramatic improvements in resource rich environments [18–21]. However, sepsis still remains as one of the major killers in South-East Asia and other resource poor settings due to inadequate capacity for implementing guidelines that are crafted for resource rich environments [22]. As a result, sub-optimal care is the norm in many healthcare facilities [23, 24]. This finding has led to a call for implementation science in care delivery to help the poorest who suffer from sepsis [24].

Like many other developing countries, service provison, capacity and quality of care in public facilities of Bangladesh are sub-optimal. A recent study conducted in 18 public facilities in Bangladesh reported that triage in the emergency departments was inadequate, infection prevention practice was poor, use of supplemental oxygen was inappropriate and administration of parenteral antibiotics for syndromic sepsis was infrequent [25]. Other studies have also reported the gaps in public facilities in terms of skilled health workforce, supply of essential drugs, equipment and logistics [26–28].

Evidence suggests that the service provision and quality of care pertaining to sepsis management in resource poor settings can be improved significantly with minimum resource allocation and investments [29–33]. Cognizant of the stark realities in Bangladesh, the International Centre for Diarrheal Diseases Research, Bangladesh (icddr,b) and the University of British Columbia in collaboration with the Ministry of Health and Family Welfare (MOHFW) of Government of Bangladesh implemented a project titled ‘Interrupting Pathways to Sepsis Initiative’ (IPSI) in rural Bangladesh between 2012 and 2015. This was a quality improvement initiative which introduced a package of interventions including a resuscitation bundle for sepsis management at select public hospitals. The objective of this paper is to describe the quality improvement process and its key outcomes.

## Materials and Methods

### Study Site and Setting

The IPSI project was implemented in 2 sub-districts of Tangail district; which is located around 120 km North West to Dhaka city, the capital of Bangladesh. The sub-districts (locally known as upazila) are Gopalpur and Bhuapur which have a total population of around 500,000. The average population density is 1432 per square km in Gopalpur and 882 per square km in Bhuapur [34]. The literacy rate is around 41% and agriculture is the main source of income. Each upazila is served by a first level referral hospital which is locally known as the Upazila Health Complex (UHC) or sub-district hospital. A sub-district hospital has around 50 inpatient beds with outdoor and emergency services.

### Study Design

We conducted a baseline environmental scan and based on the findings, we developed a simple standard operating practice for managing syndromic sepsis. Following that, we implemented a package of interventions in the demonstration hospitals, and documented the process of implementation and its outcomes between August 2013 and March 2015.

1 **Baseline environmental scan:** The baseline hospital environmental scan was undertaken to assess the structural and functional gaps pertaining to sepsis management in the demonstration hospitals. At first, we conducted a health facility survey in each hospital with trained physicians using a modified version of the WHO Health Facility Assessment Tool. Gaps related to infrastructure, availability of human resource, laboratory tests, equipment, drug and supply, knowledge and skill of health care providers, documentation and reporting system, and service utilization in outpatient, emergency and inpatient departments were explored and documented. Then, we employed a rapid hospital ethnography for five days in each facility by a team of trained physicians (#2) and an anthropologist. Gaps related to existing patient flow system, triage, admission procedure, transfer of patient to the inpatient department, initiation of treatment in the inpatient department, confidence of the health care providers, and monitoring and supervision mechanism were explored.
  The following gaps were identified specific to management of syndromic sepsis:
  - **Infrastructure:** There were no dedicated beds or areas for pediatric patients suffering from infectious diseases. Hand washing facility was inadequate and ventilation system was poor.
  - **Knowledge, skills and practice of health care providers:** There was inadequate knowledge regarding syndromic sepsis and its management options. Standard Operating Procedure (SoP) and job aids to manage syndromic sepsis were absent. There were also improper infection prevention and sanitation practices.
  - **Confidence of the health care providers:** Doctors and nurses were not confident in managing neonates and young infants. As a result, nearly all children presenting with signs of severe infections were referred to higher level facilities. Regarding neonates, nearly all of them were referred to higher level facilities from the emergency department irrespective of their presenting complaints.
  - **Equipment, drugs and logistics:** None of the hospitals had pulse oximeter or glucometer. The hospitals reported frequent stock outs of essential drugs (parenteral antibiotics, intravenous fluid) and supplies (infusion sets, oxygen, soaps).
  - **Documentation and record keeping:** Documentation and record keeping system in both the hospitals were poor as there was no checklist or clinical service register. Information regarding personal details, clinical status and treatment details were inadequately documented in individual patient record forms in the inpatient department.
  - **Patient flow system and delays:**
    - There was no functioning triage in the emergency department. During busy hours, even the critically ill children had to wait in queue for long durations.
    - There was delay in assessment and taking decisions regarding admission. During office hours (8 am to 2 pm), the emergency departments were serviced by a physician and a paramedic. However, after office hour, the physician used to stay on-call and the emergency department was primarily managed by a paramedic. Upon arrival of a sick children, the on-duty paramedic used to call the on-call physician after primary assessment. Then the on-call physician used to come to the emergency department, re-assess the child and take decision regarding admission.
    - There was delay in transferring the patient after admission, as there was no assistance in navigating the patient from the emergency to the inpatient department.
    - There was delay in initiating treatment in the inpatient department. The inpatient department was primarily managed by an on-duty nurse and an on-call physician. Upon arrival of a sick child after admission, the on-duty nurse used to call the on-call physician. The on-call physician used to come, assess and give order to the on-duty nurse to commence treatment. Moreover, there was no official mechanism to inform the inpatient from the emergency department regarding an admission.
  - **Supervision mechanism:** There was no systematic mechanism for monitoring and supervision of clinical and administrative staff.

**Fig 1:**
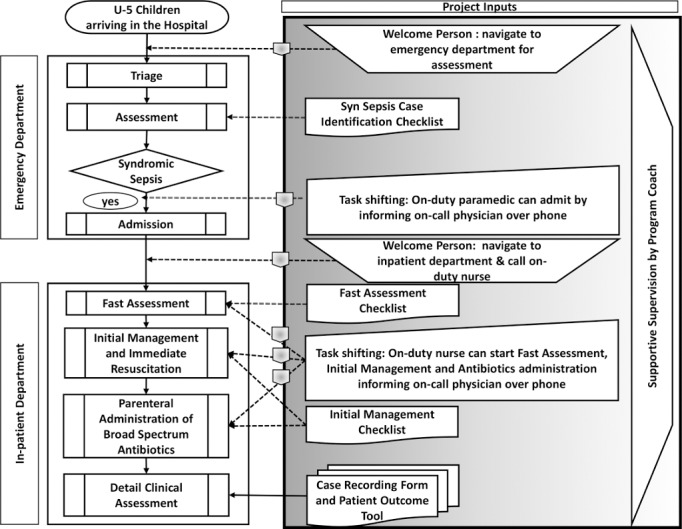
Operational flow and guideline of managing syndromic sepsis.

2 **Developing a customized action plan for Quality Improvement:** A workshop (one day) was organized in each hospital to share key findings from the baseline environmental scan. Facility managers and concerned clinical service providers (doctors, nurses and paramedics) attended the workshop and developed a customized action plan to address specific structural and functional gaps. Key recommendations to improve quality of care pertaining to management of syndromic sepsis included developing a simplified Standard Operating Procedure (SoP), education and skill enhancement of clinical service providers, developing job aids, introduction of patient record form and checklists, correction of the patient flow system, task shifting to minimize delays, introducing a supportive supervision mechanism, minor structural renovation, ensuring supply of essential equipment, drugs and logistics.
3 **Developing a simplified SoP and an Implementation Package for management of syndromic sepsis:** A simplified SoP was developed based on the Survival Sepsis Campaign guidelines [35], the Bangladesh adapted version of WHO pocket Book for Hospital Care of Children [36] and the Bangladesh National Neonatal Health Strategy and Guidelines of Bangladesh [37]. Senior pediatricians and public health specialists of Bangladesh were consulted to finalize the SoP. The SoP recommended four key steps for effective management of syndromic sepsis (Fig 1).

**Step 1- Triage, Identification and Rapid Admission:** Sick children will be prioritized in the emergency department followed by identification of syndromic sepsis through clinical assessment and immediate admission for proper treatment. Neonatal (0–28 days) syndromic sepsis was defined as the presence of any of the following sign/ symptoms—hypothermia, hyperthermia, altered mental status, convulsion, respiratory distress, umbilical infection and not feeding properly. Early childhood (29 days to 59 months) syndromic sepsis was defined as the presence of any of the following sign/ symptoms—hypothermia, altered mental status, convulsion, respiratory distress and not feeding properly.

**Step 2- Fast Assessment**: After ‘Triage, Identification and Rapid Admission’; airway, breathing and circulation of the sick child will be assessed and corrective measures should be initiated if necessary. In addition, pulse, blood pressure, temperature, O_2_ saturation, random blood sugar and weight will be measured immediately after admission.

**Step 3- Initial Management and Immediate Resuscitation:** After ‘Fast Assessment’; vascular assess should be established as early as possible. Concurrently, corrective measures will be taken immediately for hypoxemia, hypothermia, hyperthermia and hypoglycemia.

**Step 4- Parenteral administration of Broad Spectrum Antibiotics:** After ‘Initial Management and Immediate Resuscitation’; broad spectrum antibiotics must be administered parenterally immediately. Parenteral Ampicillin (50 mg/kg body weight; 8 hourly) and Gentamicin (5mg/kg body weight for neonates and 7.5 mg/kg body weight for post neonates, once daily) combination will be the first line antibiotics and parenteral Ceftriaxone will be the second line antibiotics. In case of proven sepsis, the broad spectrum antibiotics will be continued for 7–10 days. In case of early discharge due to request from the care takers, oral antibiotics can be recommended after 48 hours of admission (if there is no other complication).

4 **Education and skill enhancement of clinical service providers:** A concise training package was developed based on the SoP. Doctors, nurses and paramedics directly involved with pediatric clinical services in the demonstration hospitals received one-day training on this package. Regular refreshers trainings were organized (quarterly in year one and six monthly in year two) to reinforce education and promote knowledge retention.
5 **Flow charts to assist management:** A clinical algorithm flow chart was prepared based on the SoP. These charts were placed in prominent positions in the emergency and inpatient departments to serve as quick references for clinicians. Some additional visual reminders related to infection prevention practice, hand washing and waste management were also developed and displayed in prominent positions.
6 **Correction of patient flow system:** The patient flow system was streamlined to address the delays in triage, assessment, admission and initiation of treatment. Two specific measures were taken for this purpose.
  - **‘Welcome Persons’:** A lay person known as the ‘Welcome Person’ was positioned 24/7 in the emergency department to facilitate the patient flow system. The ‘Welcome Person’ was responsible for greeting all children after arriving in the hospital and directly take them to the clinical service provider at the emergency department for clinical assessment. After ‘Tirage, Identification and Admission’, the ‘Welcome Person’ used to navigate the syndromic sepsis cases to the inpatient department and call in the on-duty nurse for conducting ‘Fast Assessment’.
  - **Task shifting:** Selective task shifting was done to address delays related to admission in the emergency department and initiation treatment in the inpatient department. Task shifting in the emergency department ensured that the on-duty paramedic was allowed to clinically assess children, identify syndromic sepsis and admit them immediately by taking consent from the on-call physician over telephone. Similarly, task shifting in the inpatient department allowed the on-duty nurses to conduct ‘Fast Assessment’, ‘Initial Management and Immediate Resuscitation’ and ‘Parenteral administration of Broad Spectrum Antibiotics’ immediately with assent from the on-call physician.
7 **Introduction of checklists and patient record forms:** A ‘Syndromic Sepsis Case Finding Checklist’ was introduced in the emergency department. The checklist was filled by the on-duty paramedic for all children presenting in the emergency department. ‘Syndromic Sepsis Fast Assessment Checklist’, ‘Initial Management Checklist’, ‘Case Recording Form’, and ‘Management Outcome Tool’ were introduced in the inpatient department. These tools and checklists were maintained for all children admitted in the inpatient department with syndromic sepsis. These checklists were followed and filled by the on-duty nurse in the inpatient department.
8 **Minor structural renovations:** In the emergency department a hand washing facility with liquid soap dispenser was installed and minor repairs were made to improve infection control measures. In the inpatient department, a separate pediatric area to segregate children with suspected infections was created. The ventilation system was improved and hand washing facilities were established. Infection control measures also included basic medical waste management training which was provided to all concerned health care providers. Basic steps included- waste minimization, segregation, proper handling and disposal of waste.
9 **Equipment and supply chain management:** Glucometers and pulse oximeters were provided to each hospital. Intravenous fluids, injectable antibiotics (Ampicillin, Gentamicin and Ceftriaxone), infusion sets and syringes were supplied to prevent stock-outs. The hospital managers and supply chain personnel were advised on how to maintain an updated inventory of essential medications and fluids.
10 **Supportive supervision and Program Coach:** To ensure comprehensive implementation of the intervention package, central and onsite supportive supervision were introduced. Central supervision was done by key personnel from the Directorate General of Health Services through periodic field visits. Each of the demonstration hospitals was served by a physician employed by the project as a ‘Program Coach’. The ‘Program Coach’ provided on site supportive supervision during office hours and remained on-call after office hours to provide technical support to the clinical staff. The ‘Program Coach’ used to review the checklists and patient record forms, identify potential gaps regarding delays and adherence to SoP, and provide feedback to the concerned clinical staff and hospital managers to overcome those gaps.

### Data Collection & Extraction

Data was extracted from the ‘Case Identification Checklist’, ‘Fast Assessment Checklist’, ‘Initial Management Checklist’, ‘Case Recording Form’ and ‘Management Outcome Tool’ by project staff who were not involved with implementation. After taking necessary preparation, the simplified SoP was officially launched in the demonstration hospital on 22 August, 2013. We are presenting the information from the launching till 31 March, 2015.

### Ethics Statement

This was a quality improvement initiative which adopted national and international guidelines for managing syndromic sepsis. The service was delivered through government system as a routine service. Necessary approval was obtained from the Directorate General of Health Services of Ministry of Health and Family Welfare before commencing the quality improvement process in demonstration hospitals. In addition, we also obtained approval from the Institutional Review Board of icddr,b which consists of two independent committees; i.e Research Review Committee and Ethics Review Committee (protocol number PR 12076). We only extracted data from routine registers and hospital patient record forms with necessary de-identification. We did not interact with patients for collecting additional data for the study. Hence, the Institutional Review Board of icddr,b and the Directorate General of Health Services exempted us from obtaining consents from individual patients.

### Data Analysis

Data was entered and stored in Microsoft SQL server and STATA version 13 special edition (College Station, Texas, USA) was used to analyze data. We used descriptive statistics to present the quality of care related to key steps of managing syndromic sepsis as per the SoP. Hypoxemia was defined as having pulse O_2_ saturation below 92%. Hypothermia was defined as having axillary temperature below 96°F and hypoglycemia as serum blood sugar level below 2.5 mmol/L. Each indicator was stratified by: age (0–28 days as neonates and 29 days-59 months as post neonates) and sex (male and female), hospital (Gopalpur and Bhuapur), time of admission (8.31 am-2 pm as office hours and 2.01 pm-8.30 am as after office hours) and day of arrival (Saturday-Thursday as weekdays and Friday as weekends). For categorical variables, we estimated proportions and for continuous variables, we estimated means and standard deviations. The point estimates were reported with 95% CI. Inferential statistics were used to assess the difference in proportions or means between categories of stratifying variables.

Time gaps between emergency arrival and completion of key steps related to managing syndromic sepsis were assessed. We used median with the inter-quartile range to present the time gaps as distributions of the data was skewed (checked using Kernel density estimation). The Mann-Whitney U test was employed for the equality of median for continuous variables. All the statistical tests were carried out at 5% level of significance with two sided alternatives.

## Results

Between August 2013 and March 2015, a total of 1262 children (648 in Bhaupur and 614 in Gopalpur) were identified as syndromic sepsis in the emergency departments. Among the neonates identified as syndromic sepsis (n = 208), the most common sign/symptom was respiratory distress (72%) followed by feeding problem (43%), altered mental status (28%), hypothermia (11%), umbilical infection (9%), hyperthermia (8%) and convulsions (6%). Similarly, among the post neonates, the most common sign/symptom was respiratory distress (76%), followed by convulsions (23.6%), feeding problem (17%), altered mental status (13.6%) and hypothermia (3%).

Of the 1,262 children identified as syndromic sepsis, 82% were admitted, 10% were referred to the higher level district hospital from the emergency department and 8% refused admission. The rate of referral was much higher among neonates than post neonates (30% vs 6%; p<0.05). The rate of referral among neonates did not vary significantly during the implementation period. Among the neonates who were referred (N = 62), the main presenting sign/symptoms were convulsion (67%), altered mental status (53%) and not feeding properly (38%). Among the post neonates who were referred (N = 62), the chief presenting complaints were hypothermia (26%) and altered mental status (23%). Regarding those who refused admission, umbilical infection (33%) was the predominant sign among neonates and hypothermia (23%) among post neonates. The rate of referral was higher among females (13%) than males (6%) (p<0.05). No significant difference was noted in rate of referral by hospital, time of arrival and day of arrival.

Table 1 summarizes quality of care related to ‘Fast Assessment’ of syndromic sepsis cases. Almost all the neonates and post neonates had their blood oxygen saturation level, blood glucose level, pulse rate, temperature and weight measured immediately after admission in the inpatient department. There was no significant variation among the stratifying variables. However, only one third had their blood pressure measured with significant variation between age (neonate 3% and post neonate 38%; p<0.005), hospitals (Bhuapur 8% vs Gopalpur 62%; p<0.005) and time of arrival (office hours 29% vs after office hours 38%; p<0.05).

**Table 1:** Status of Fast Assessment of syndromic sepsis patients in the inpatient department.

		admitted	Measured/performed					
			O2 sat	RBS	Temp	Pulse	BP	Wt (kg)
		N	%	%	%	%	%	%
Age	Neonate	134	100.0	98.5	100.0	100.0	3.0*	100
	Post-neonate	902	99.4	99.4	100.0	100.0	38.1*	100
Sex	Male	690	99.4	99.4	100.0	100.0	32.0	100
	Female	346	99.7	99.1	100.0	100.0	36.7	100
Hospital	Bhuapur	544	100.0	98.9	100.0	100.0	7.5*	100
	Gopalpur	492	99.0	99.8	100.0	100.0	62.4*	100
Time of arrival	Office hours	484	99.4	98.8	100.0	100.0	28.9ⱡ	100
	After office hours	550	99.6	99.8	100.0	100.0	37.5ⱡ	100
Day of arrival	Weekdays	913	99.5	99.2	100.0	100.0	32.6	100
	Weekend	123	100.0	100.0	100.0	100.0	40.7	100
	Total	1036	99.5	99.3	100.0	100.0	33.6	100

* = p<0.005

ⱡ = p<0.05

**Fig 2:**
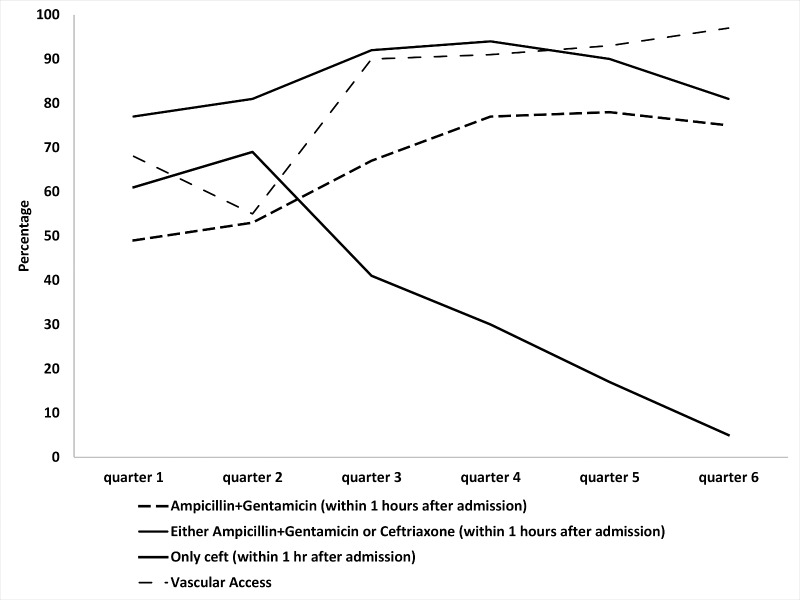
Quarter wise trend in the vascular access and use of first dose of parenteral antibiotics among syndromic sepsis patients in the inpatient department (August 2013-March 2015).

Table 2 presents the key findings regarding ‘Initial management and Immediate Resuscitation’ of syndromic sepsis patients in the inpatient department. One fifth of the syndromic sepsis cases had hypoxemia during admission. More neonates (37%) were hypoxemic than post neonates (18%) (p<0.005). Females were more likely to have hypoxemia (26%) than males (18%) (p<0.005). Almost all (94%) of the hypoxemic children received supplemental oxygen immediately without significant variation among different strata. Very few had hypoglycemia (0.3%) or hypothermia (0.5%) during admission. Vascular access was established in 79% of the admitted cases (Fig 2) with significant improvement between the first quarter (55%) and the last quarter (97%) (p<0.5).

**Table 2:** Status of Initial Management and Immediate Resuscitation of syndromic sepsis patients in the inpatient department.

		Admitted	Hypoxemia		Hypoglycemia		Hypothermia		Vascular Channel Establishment	AB given (IV/IM)
			Identify	O2 given	Identify	Glucose given	Identify	Measure taken		
		N	%	%	%	%	%	%	%	%
Age	Neonate	134	37.3*	94.0	0.7	0.0	3.0*	100	78.4	97.8
	Post-neonate	902	18.4*	94.0	0.3	66.7	0.1*	100	78.5	97.3
Sex	Male	690	18.4*	94.5	0.4	33.3	0.6	100	79.9	97.5
	Female	346	25.7*	93.3	0.3	100.0	0.3	100	75.7	97.1
Hospital	Bhuapur	544	20.2	96.4	5.7	66.7	0.0	100	85.7*	98.7
	Gopalpur	492	21.5	91.5	0.2	0.0	1.0	100	70.5*	95.9
Time arrival	Office hours	484	21.1	93.1	0.4	50.0	0.7	100	77.5	97.3
	After office hours	550	20.5	94.7	0.3	50.0	0.4	100	79.3	97.5
Day of arrival	Weekdays	913	21.0	93.8	0.4	50.0	0.6	100	78.6	97.3
	Weekend	123	19.5	95.8	0.0	0.0	0.0	100	77.2	98.4
	Total	1036	20.8	94	0.4	50.0	0.5	100	78.5	97.4

* = p<0.005

Around 85% of the syndromic sepsis cases received the first dose of recommended antibiotics parenterally (Ampicillin + Gentamicin in combination or Ceftriaxone) within one hour of admission (Fig 2). Approximately 90% received the first dose of recommended antibiotics in the first three hours after admission. More neonates (76%) received the recommended antibiotics than post neonates (62%) within one hour of admission; (p<0.05).

Fig 2 demonstrates the quarterly trend in administration of the first dose of recommended antibiotics parenterally within one hour of admission. There was significant improvement in choice of the first line recommended antibiotics i.e Ampicillin and Gentamicin in combination (from 49% to 75%; (p<0.005) during the implementation period. Similarly, choice of the second line recommended antibiotic i.e Cenftriaxone decreased from 61% to 5%, (p<0.005) between the first quarter and the last quarter.

Table 3 demonstrates the time gap between arriving in the emergency department and conducting key steps related to management of syndromic sepsis. The median time taken to complete the assessment and identify sysdromic sepsis in the emergency department was 6 minutes with an inter quartile range of 5–15 minutes, i.e assessment and identification was completed within 5 minutes for 25% of the patients and within 15 minutes for 75% of the patients. Similarly, the median time taken complete the ‘Fast Assessment’ was completed 15 minutes (inter quartile range 6–20 minutes). ‘Initial Management and Immediate Resuscitation’ was completed with a median value to 35 minutes (inter quartile range 25–45 minutes). Similarly, the median value between arriving in the emergency department and receiving the first dose of parenteral antibiotics was 35 minutes (inter quartile range 25–45 minutes). All of these four steps of managing syndromic sepsis was faster in Bhuapur than Gopalpur (p<0.05). However, completion of ‘Initial Management and Immediate Resuscitation’ and ‘Parenteral administration of first dose of antibiotics’ were significantly slower among neonates than post neonates (median 40 minute vs 35 minutes for both steps, p<0.05).

**Table 3:** Time gaps between arriving in the emergency department and conduction of different steps of management of syndromic sepsis.

		Median (IQR) time gap (in mins) between arrival at the emergency and _			
		Identification and Rapid Admission	Fast Assessment	Initial management and Immediate Resuscitation (start)	1st dose AB
Age	Neonate	8.5(5–15)	15(10–20)	40(30–50)*	40(28–50)*
	Post-neonate	6(5–15)	15(5–20)	35(25–45)	35(23–45)
Sex	Male	5(5–15)	15(5–20)*	35(25–45)	35(20–45)
	Female	9.5(5–15)	15(10–20)	35(25–45)	35(25–45)
Hospital	Bhuapur	5(5–8)*	10(5–15)*	25(20–35)*	25(18–35)*
	Gopalpur	15(5–20)	20(15–25)	40(35–50)	40(35–50)
Time of arrival	Office hours	6(5–15)	15(10–20)*	35(25–45)	35(25–45)
	After office hours	7(5–15)	15(5–20)	35(23–45)	35(20–45)
Day of arrival	Weekdays	6(5–15)	15(5–20)	35(25–45)	35(25–45)
	Weekend	7(5–15)	15(10–21)	35(23–45)	35(22–45)
	Total	6(5–15)	15(6–20)	35(25–45)	35(25–45)

* = p<0.05

**Fig 3:**
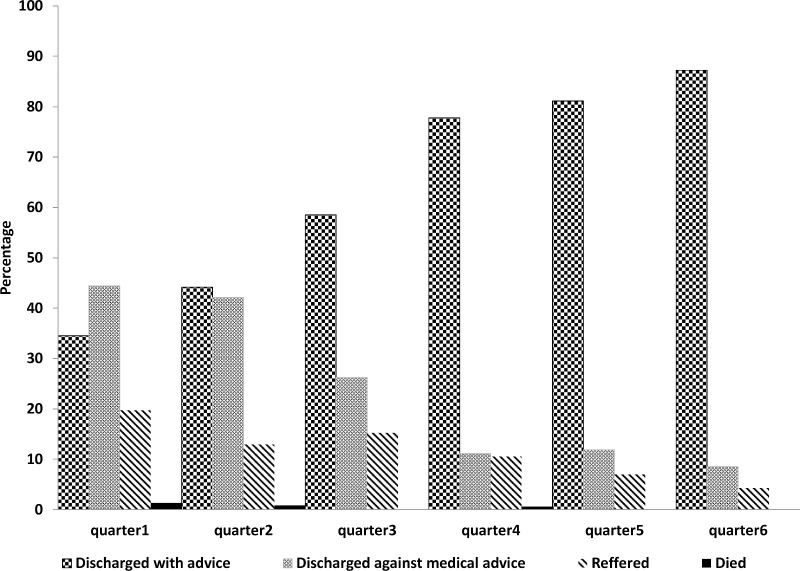
Quarter wise trend in hospital outcome of syndromic sepsis patients (August 2013-March 2015).

Fig 3 shows the quarterly trend in hospital outcomes of patients admitted with syndromic sepsis. In the first quarter of implementation (August-December 2013), 35% patients were discharged with medical advice. The condition improved significantly in the last quarter (January-March 2015) as more than 85% were discharged with medical advice. Around 20% patients admitted with syndromic sepsis were referred to a higher level facility in the first quarter, which decreased to less than 5% in the last quarter. There were only three inpatient deaths among all children admitted with syndromic sepsis. The average duration of hospital stay was 2.2 days with a median value of 2 days (inter quartile range 1–3 days). In the first quarter, 47% patient with syndromic sepsis stayed in the facility for more than 48 hours as opposed to 80% in the last quarter. The percentage of children leaving the facility in less than 24 hours decreased from 29% to 4% between the first and the last quarter.

## Discussion

This quality improvement project was implemented because of inadequate service readiness and poor care delivery for managing neonatal and early childhood syndromic sepsis in the demonstration hospitals. The full intervention package was designed based on local contexts and resources, and in such a manner that after a sick child arrives in the emergency department, the hospital and healthcare providers were prepared to rapidly identify and manage syndromic sepsis as per the SoP. Service availability and quality of care pertaining to management of syndromic sepsis in the demonstration hospitals were improved and sustained over the implementation period. The two participating sub-district hospitals were representative of similar facilities in resource constrained settings.

In addition to the traditional approach of quality improvement through training alone, we tested innovative approaches which may have played decisive role in addressing the existing gaps and changed practices within a short period. This began with the use of a ‘Baseline Environmental Scan’ to assess the needs and prioritize interventions addressing specific gaps. The action plan for improving service availability and quality of care was primarily designed and implemented by the hospital managers and concerned clinical staff. As a general principle, the project played a supporting role in the quality improvement process, which played a cardinal role in developing ownership regarding the overall implementation package. The novel approach of introducing a ‘Welcome Person’, as well as ‘Task Shifting’ contributed to reducing the documented delays in triage, assessment and treatment, including high adherence to the SoP. Onsite supportive supervision by the ‘Program Coach’ and organizing onsite regular refreshers trainings also have led to improved clinical practices and sustaining the improved quality of care.

Early and appropriate care seeking is one of the most important aspects for interrupting the pathways to deaths due to syndromic sepsis. Neonates and young infants are innately vulnerable to severe infection leading to sepsis. Angus et al. (2001) reported that incidence of sepsis among infants (<1 year of age) is almost 9–10 times higher than older children (1–4 years and 5–14 years) [38]. On an average, the emergency department of each hospital was visited by only 6 neonates with syndromic sepsis monthly. Considering a catchment population of around 250,000 for each of the demonstration hospitals and innate vulnerability of neonates, the number seems to be very low. The most likely explanation could be-lack of education of parents regarding danger signs leading to inappropriate care seeking practices as seen in rural settings of low income countries [39–43]. In some parts of rural Bangladesh, the traditional practice is to confine movement of mothers and newborns outside home during the early neonatal period [44]. This may delay the care seeking practice until the neonate becomes severely ill, an assertion which is supported by the findings of our study where a greater proportion of neonates presented with syndromic sepsis had severe symptoms like hypoxemia and hypothermia.

The first step of managing syndromic sepsis was to ensure ‘Triage, Identification and Rapid Admission’ of children with syndromic sepsis. The baseline environmental scan revealed that the emergency departments of the demonstration facilities did not have a functioning triage or a structured system to assess and identify syndromic sepsis. Almost all the newborns and young infants were referred to higher level facilities irrespective of their presenting complaints as the clinical service providers felt less confident in treating neonates through inpatient services. In support to this explanation, a recent survey conducted in 12 first level public facilities of Bangladesh also reported that the proportional contribution of neonates among all inpatient admissions is less than 1% [25]. Given that neonates are infection prone [38], this `ure affirms the reluctance towards admitting neonates at first level hospitals. We were reasonably successful with our approach as around four fifths of the children identified as syndromic sepsis were admitted in the inpatient department. Among the neonates, around 30% were referred out after introducing the quality improvement initiatives. Although the rate of referral among neonates was still on the higher side, it was a significant improvement from the previous practice where almost all newborns were referred out from the emergency department. This also implies that with introduction of triage and systematic assessment in the emergency department supported by onsite supervision by ‘Program Coaches’, the clinical service providers became more confident in treating neonates through inpatient services. Though we have tried to further reduce the rate of referral among neonates through training and supportive supervision, the rates did not vary significantly over the implementation period. We do not have conclusive information regarding the appropriateness of these referral, but the relative higher rate among neonates can be explained by their innate vulnerability and lack of readiness of the first level facilities to manage newborns with major complications. The need for further research to explore contributing factors and potential solutions to this problem is emphasized through this finding.

The next steps in managing syndromic sepsis are to ensure ‘Fast assessment’ followed by ‘Initial Management and Immediate Resuscitation’ in the inpatient department. With some pitfalls, most of the children with syndromic sepsis were assessed quickly and the resuscitation bundle was initiated immediately. These changes further support the findings and recommendation from other studies which have reported the improvement in quality of care through capacity development and with minimum investments in resource poor setting [29–33]. Apart from the sepsis education through refreshers trainings and supportive supervision by onsite ‘Program Coaches’, ‘Task Shifting’ to nurses have contributed significantly in changing the existing practice, adhering to the guidelines and sustaining high standard of care. Other studies have also reported that nurses have less turnover rate than doctors, demonstrate higher motivation and possess better retention of skills [45]. Although almost all of the admitted children with hypoxemia received immediate oxygenation through traditional practice, the high rate of hypoxemia (21%) signifies the importance of introducing bubble CPAP, BiPAP in similar settings [46–48].

Early administration of broad spectrum antibiotic parenterally is one of the most important steps in management of syndromic sepsis. The Surviving Sepsis Campaign Guideline recommends administering the first dose of antibiotic within one hour of admission [35]. Approximately 85% of the children with syndromic sepsis received the first dose of recommended antibiotic parenterally within one hour of arrival. Around three quarters of them received the first dose within 45 minutes of arrival; an achievement that has eluded practitioners even in resource rich areas. Several factors including facilitation of triage through the ‘Welcome Persons’ and ‘Task Shifting’ in the emergency department, introduction of protocolized management, ‘Task Shifting’ in the inpatient department and on site supportive supervision by ‘Program Coaches’ may have been influential in reducing possible delays in administering key steps of sepsis management. The novel approach of introducing a ‘Welcome Person’ to navigate patients to appropriate service delivery points, facilitate the patient transfer process from the emergency to the inpatient department, and maintaining liaison with clinical service providers may have played the most catalytic role in this regard. The attribution and effectiveness of the ‘Welcome Person’ in improving quality of care and reducing delays need further assessment and understanding.

The National Neonatal Health Strategy and Guidelines of Bangladesh (2009) and the Bangladesh adapted version of WHO Pocket Book for Hospital Care of Children recommend using the combination of Ampicillin and Gentamicin as the first line antibiotics for managing syndromic sepsis [36, 37]. The combination is chosen over the Ceftriaxone as it is safer, cheaper and has high synergistic bactericidal effect [46, 49]. The choice for first line antibiotic shifted from Ceftriaxone to the combination of Ampicillin and Gentamicin in both facilities over the project implementation period. We believe this change in practice was the result of continuous supportive supervision and repeated feedback loop by onsite ‘Program Coaches’. Considering the higher cost, side effects and possible resistance to Ceftriaxone, the shift in choice and practice has larger policy implication [46].

Duration of hospital stay is one of the main concerns for effective management of syndromic sepsis. Although, there was some improvement over time, the average duration of hospital stay was still around only 3 days in the last quarter. Cultural barriers and financial constraints due to high out of pocket expenditure and opportunity cost during hospital stay could explain the behavior [50, 51]. The ‘Program Coaches’ encouraged the clinical service providers to counsel the parents to stay for longer duration and complete the treatment. The focus was to ensure at least 48 hours of hospital stay as the recent WHO recommendation suggests that 2 days of parenteral antibiotic followed by 5–7 days of oral antibiotic is safe and effective [52–54]. With the change in approach and counselling of parents by clinical service providers, there was significant improvement in more than 48 hours of hospital stay over time. Acknowledging the challenge of ensuring 7–10 days of hospital stay in resource poor settings like Bangladesh, focusing on the first 48 hours can be an important program learning for other projects in similar settings.

Though the project has demonstrated reasonable accomplishment related to implementation of most of the key components of sepsis management, the rate of establishing vascular access did not reach a high standard. This is similar to other study findings as a meta-analysis of 128 literature published between 1975 and 2011, reported that first attempt to establish an intravenous channel fails in 12–26% of adults and 24–54% of children [55]. Factors associated with this failed attempts could be the characteristics of patients (age, weight, skin color, etc.), insertion site, catheter caliber and operator's expertise, etc. [55, 56]. After a few failed attempt the procedure becomes increasingly difficult as the child becomes more anxious and fearsome which in turn raises the sympathetic activity causing vasoconstriction. Furthermore the inability to establish an intravenous channel also creates a negative impact on the nursing staff which may have decreased their self-confidence and therefore intended efficiency [57]. This signifies the importance of introducing additional aid (eg. ACCU vein) in establishing intravenous channel and intraosseous channels can be tested as they have shown encouraging results in other settings [57, 58]. There was a difference between the hospitals in measurement of BP as only 8% of the admitted cases in Bhuapur had their BP measured in contrast to 62% in Gopalpur. On the other hand, the level of use of recommended antibiotic was higher in Bhuapur than Gopalpur. We do not regard low levels of BP measurement as a major oversight in care because in children BP measurements are difficult and also hypotension is a late sign in children and is rarely used to diagnose shock even in resource rich settings. However, the contrasting performance of the two demonstration hospitals regarding some indicators underpins an important program learning. Consistent with our study findings, other initiatives have also acknowledged that even with the same level of inputs, uptake and sustainability of a generic set of interventions can be different in different context [59, 60]. This signifies the importance of developing context specific action plan for implementing a generic guideline or intervention package.

The quality improvement initiatives were undertaken in only two demonstration sites and the findings presented in this paper may lack external validity to be generalizable nationally and regionally. However, the inadequate service readiness and poor quality of care that were existent in the demonstration hospitals before introducing the intervention package are mostly generalizable to other public hospitals in resource constrained settings. Therefore, the findings from this paper present important program learnings to be tried and tested in similar settings. One of the other limitations of this initiative is the inability to attribute different components of the intervention package to different achievements. We can only infer that the interventions have worked as a package but cannot report the attributable fraction of an individual innovation and initiative. The prime objective was to improve the quality of care in the shortest possible time. The intervention package was updated and upgraded throughout the implementation period through the ‘Pan-Do-Study-Act’ cycle [61]. Therefore, the package may have been ‘over-dosed’ rather than ‘under-dosed’. Though we do not have extensive data regarding costing, the package focused on addressing gaps related to service readiness and capacity of health workforce with minimum external inputs. Regarding service readiness, the package focused on ensuring supply of equipment, drugs and logistics essential for managing syndromic sepsis. All of them are already included in the essential list of equipment, drug and logistics for first level facilities as recommended in the National Neonatal Health Strategy and Guidelines of Bangladesh (2009) (37). Government of Bangladesh has already established a Quality Improvement Secretariat under the Ministry of Health with specific focus on maternal and newborn health. The secretariat can replicate the role of ‘Program Coaches’ to provide supportive supervision and necessary oversight to improve quality of care. Regarding streamlining the patient flow and reducing delays, we believe that the maximum return was achieved from the welcome person which is a low cost intervention. The welcome person can also take the responsibility of navigating all maternal and newborn cases in a hospital to reduce possible delays and improve quality of care. Considering the minimum investment that is required to address the structural and functional gaps and national priority to improve quality of care, we strongly believe that the package has great potential to sustain at scale. However, further refinement can be considered to make the intervention package less resource intensive and more cost effective before potential scale up. Another important aspect of this quality improvement initiative is its potential to reduce the burden of inappropriate referral to higher level facilities which can further reduce the cost on health systems. The other limitation related to inference of these findings is the absence of specific estimates from baseline or from a comparison hospital. Since this was a quality improvement initiate, the major focus was on improving the processes over time and achieve standard quality of care benchmarks. Most of these practices and services were not available in the demonstration hospitals, and hence specific estimates for comparison were not available. However, the ‘Baseline Environmental Scan’ documented the poor preparedness and inappropriate sepsis management practices in the demonstration hospitals. The visible improvement in quality of care can therefore be linked to the intervention package introduced by this project. The other limitation is the effect of possible reporting bias as the on-duty paramedics and nurses were responsible for documenting the steps of managing syndromic sepsis through the checklists and patient record forms. However, the effect may have been minimized due the continuous presence of ‘Welcome Persons’ and supportive supervision by on-site ‘Program Coaches’. The Hawthorne effect [62, 63] could have been another limitation of our study but it is very unlikely that this effect would persist with almost two years of implementation.

## Conclusion and Recommendation

With minor limitations, quality of care pertaining to sepsis management can be improved with a well-designed, need based and context specific intervention package even for the most resource limiting environment. Successful testing of some novel approaches such as facilitation support for patient navigation (Welcome Person) and onsite supportive supervision (Program Coach) to maintain PDCA (Plan→ Do→ Check→Act) cycle are important program learnings.
